# Real-world outcomes of ^177^Lu-PSMA-I&T in metastatic castration-resistant prostate cancer: the KuPSMALu trial in Eastern Finland

**DOI:** 10.2340/1651-226X.2025.44748

**Published:** 2025-11-11

**Authors:** Okko-Sakari Kääriäinen, Pekka Poutiainen, Heidi Gröhn, Timo Voivalin, Hanna Mussalo, Satu Pukkila, Kirsi Ketola, Päivi Auvinen

**Affiliations:** a Department of Oncology, Kuopio University Hospital, the Wellbeing Services County of North Savo, Kuopio, Finland; b Institute of Clinical Medicine, University of Eastern Finland, Kuopio, Finland; c Diagnostic Imaging Center, Kuopio University Hospital, Kuopio, Finland; d A.I. Virtanen Institute for Molecular Sciences, University of Eastern Finland, Kuopio, Finland; e Institute of Biomedicine, University of Eastern Finland, Kuopio, Finland

**Keywords:** Castration-resistant prostate-cancer, PSMA-PET, ^177^Lu-PSMA radioligand therapy, theranostics

## Abstract

**Background and purpose:**

Radioligand therapy targeting prostate-specific membrane antigen (PSMA) with lutetium-177 PSMA (¹⁷⁷Lu-PSMA) compounds has emerged as an effective treatment for metastatic castration-resistant prostate cancer (mCRPC). The KuPSMALu trial evaluated the real-world efficacy and safety of in-house produced ¹⁷⁷Lu-PSMA imaging & therapy (I&T) for mCRPC patients in a public healthcare setting and assessed whether selection based on ¹⁸F-PSMA-PET and contrast-enhanced CT – without FDG-PET – provides favourable oncological outcomes.

**Patients/material and methods:**

This prospective, single-centre observational study included 40 patients with PSMA-positive mCRPC who had progressed after chemotherapy and at least one androgen receptor pathway inhibitor. Patients received 3–6 cycles of ¹⁷⁷Lu-PSMA-I&T at 6–8-week intervals. Imaging, blood-based markers and patient-reported outcomes were collected longitudinally. Dosimetry, adverse events (AEs) and quality-of-life metrics were systematically assessed.

**Results:**

The median overall survival (mOS) was 16.0 months. ECOG 0–1 patients had significantly longer mOS than ECOG 2 patients (20.0 vs. 4.7 months, *p* < 0.01). A PSA decrease ≥ 50% was observed in 40% of patients and correlated with improved mOS (23.7 vs. 9.1 months, *p* < 0.01). PSA doubling time (dt) > 4 months predicted superior survival (23.8 vs. 12.6 months, *p* = 0.040). Grade ≥ 3 AEs occurred in only 12.3% of patients.

Interpretation: In-house ¹⁷⁷Lu-PSMA-I&T production combined with pragmatic imaging-based patient selection provides a safe, cost-effective therapy for mCRPC in public healthcare. PSA kinetics, particularly PSA dt, are strong predictors of therapeutic benefit. The findings align with VISION and TheraP trials and highlight the feasibility of integrating radioligand therapy into routine clinical care.

## Graphical abstract

**Figure:**
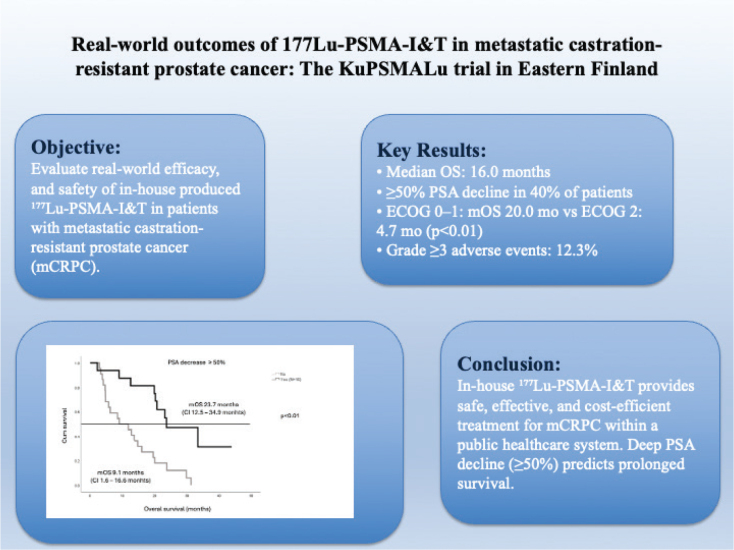

## Introduction

Prostate cancer is the second most common malignancy in men worldwide, surpassed only by lung cancer in incidence. In terms of cancer-related mortality, it stands as the fifth leading cause of death among men [1]. The prognosis for localised prostate cancer is generally favourable, with over 90% of patients achieving curative outcomes. The risk of metastases increases with elevated prostate-specific antigen (PSA), positive surgical margins and high International Society of Urological Pathology (ISUP) grade [2]. Metastatic prostate cancer (mPC) remains incurable, presenting initially as hormone-sensitive disease (mHSPC) and later as castration-resistant disease (mCRPC). Patients with high-volume disease and synchronous metastases have a worse prognosis [3].

Significant therapeutic advances have been made over the past two decades. Androgen deprivation therapy (ADT), using luteinizing hormone-releasing hormone (LHRH) agonists, antagonists or orchiectomy, has long been the cornerstone of treatment for mPC. The introduction of the chemotherapeutic agents of docetaxel and cabazitaxel, has led to improved outcomes at multiple stages of mPC [4–6]. Furthermore, new androgen receptor pathway inhibitors (ARPis) such as abiraterone [7–9], enzalutamide [10, 11], apalutamide [12] and darolutamide [13] have provided significant gains in progression-free survival (PFS) and overall survival (OS) and are well-tolerated in clinical practice. In addition, poly (ADP-ribose) polymerase (PARP) inhibitors, such as olaparib [14] and talazoparib [15], represent promising targeted therapies for patients with BRCA1/2 and other homologous recombination repair (HRR) mutations.

Radionuclide therapy (RLT) has improved OS and symptom control. Radium-223 [16] and samarium-153 [17] incorporate into newly formed bone matrix at osteoblastic metastatic lesions and both have demonstrated benefits for painful bone metastases. In the ALSYMPCA trial, radium-223 also prolonged mOS [16]. Owing to its mechanism of action, radium-223 is effective in patients with bone-only disease, but its effects on visceral and lymph-node metastases are minimal. Furthermore, the reported increase in bone fracture incidence among patients treated with radium-223 in combination with abiraterone and corticosteroids without the use of bone-protecting agents (BPA), has prompted caution within the scientific community regarding the safety of this regimen. [18]. The combination of radium-223 and enzalutamide with BPA has been shown to be safe for the bone health and effective in selected mCRPC patients [19].

Prostate-specific membrane antigen (PSMA) is overexpressed, particularly in mCRPC [20]. These PSMA-positive prostate cancer cells can be detected with PET imaging using 18F- or 68Ga- labelled PSMA-PET-CT [21]. ^177^Lu is a radioactive isotope that decays emitting beta particles which can cause DNA double strand brakes when delivered to cells. By combining ^177^Lu (via a chelator part) with a PSMA binding motif and linker, ^177^Lu can be delivered to PSMA-positive cells [22]. In the VISION trial, the addition of ^177^LuPSMA-617 to standard of care prolonged mOS by approximately 4 months, and ^177^Lu-PSMA-617 has been approved by the EMA and FDA for treating mCPRC patients whose disease has progressed after taxane-based chemotherapy and ARPi and whose disease is PSMA-positive in PSMA-PET [23]. In addition, ^177^Lu-PSMA-617 has demonstrated favourable tolerability compared with chemotherapy [24].

The aim of the KuPSMALu trial was to evaluate the efficacy and safety of ^177^Lu-PSMA therapy in mCRPC patients in a real-world (RWE) -trial. In addition, the study assessed whether including PSMA-positive patients using 18F-PSMA-PET and contrast-enhanced body CT, without FDG-PET, would result in favourable oncological outcomes. The radiopharmacy unit at Kuopio University Hospital (KUH) enabled in-house production of RLT tracers. Production of ^177^Lu-PSMA- imaging & therapy (I&T) began in March 2020, allowing mCRPC patients in Eastern and Nordic Finland to receive ^177^Lu-PSMA therapy as part of public healthcare services. The treatment protocol was specifically designed to ensure that the cost of ^177^Lu-PSMA-I&T would remain sustainable within the public healthcare system.

## Patients/material and methods

### Study design and patient population

This is an observational single-arm, single-centre RWE-trial, with prospectively collected data. The study included the first 40 patients, who were clinically selected to be receive ^177^LuPSMA-I&T at KUH. All patients provided written informed consent, and the trial was conducted in accordance with the Declaration of Helsinki and Good Clinical Practice. The protocol was approved by the ethics board of Eastern Finland and by the KUH.

Eligible patients were required to have metastatic castration-resistant prostate cancer (serum testosterone level below 1.7 mmol/L) with evidence of disease progression following treatment with an ARPi and taxane-based chemotherapy. Patients deemed unsuitable for further chemotherapy due to clinical factors or those who declined chemotherapy, were also eligible for inclusion, provided they had experienced disease progression on at least one ARPi therapy. Patients had to have a life expectancy of at least 6 months, as estimated by the treating physician. Enrolment criteria included the presence of metastatic disease beyond the skeletal system. There were no restrictions on the maximum number of prior treatment lines. To qualify, patients were required to have an Eastern Cooperative Oncology Group (ECOG) performance status of 0 to 2, while those with an ECOG performance status greater than 2 were excluded.

Exclusion criteria included impaired renal function (estimated glomerular filtration rate [eGFR] < 40 mL/min), haemoglobin (Hb) < 100 g/L, platelet count < 100×10⁹/L, white blood cell count < 3.0×10⁹/L and neutrophil count < 1.0×10⁹/L.

### Screening

All patients underwent PSMA-PET and contrast-enhanced body CT imaging to confirm the presence of PSMA-positive disease and the absence of PSMA-negative disease. For this study, ^18^F-PSMA-1007 or ^18^F-DCFPyL were used as PSMA tracers. Metastases were defined as PSMA- positive if SUVmax exceeded the SUVmax of the liver or spleen, depending on the tracer used. Patients with PSMA-negative bone metastases with a soft-tissue component were excluded from the trial. Patients with PSMA-negative visceral metastases or PSMA-negative lymph-node metastases were not eligible for inclusion. Imaging studies were evaluated in a local multidisciplinary team (MDT) meeting including an oncologist and a nuclear medicine physician. Other prostate cancer treatments, besides ADT and bone health agents (BHA), were not permitted during the ^177^LuPSMA-I&T treatment period.

### ^177^Lu-PSMA-I&T production

Production of ¹⁷⁷Lu-PSMA-I&T was carried out at KUH using the miniAllinOne (miniAIO) automated module from Trasis. The precursor, reagents (UltraPure water, ascorbate buffer and 0.9% NaCl), single-use cassettes, 25-mL sterile product vials and 25-mm syringe filters (0.2-μm Supor Membrane) were sourced from Trasis. The precursor was stored at −20°C in single-use vials, while the reagent kits and cassettes were kept at room temperature. Lutetium-177 chloride (EndolucinBeta) was acquired from ITG and stored at room temperature, with all chemicals used without further purification.

Synthesis was performed using low bioburden, single-use cassettes and reagents provided, in compliance with Good Manufacturing Practice (GMP) regulations. Two prefilled vials were connected to the cassette system prior to synthesis: the first vial contained ^177^LuCl₃, and the second contained the precursor dissolved in 2 mL of aqueous ascorbic acid buffer at pH 4.5. The contents of vial 1 were transferred into the reactor vial, followed by the contents of vial 2 via vial 1. The labelling reaction took place under controlled conditions at 120°C for 100 s, followed by 105°C for 800 s. Upon completion, the reaction mixture was diluted with isotonic saline and filtered through a sterilising 0.2-μm membrane filter.

Productions were carried out according to the EU-GMP protocol in clean-room conditions. For each batch, several quality control parameters were assessed, including chemical and radiochemical purity, solvent residues, colloidal lutetium, pH, endotoxins and sterility. The yield was quantitative, and the product purity was 100%. Typically, two patient doses were prepared per production, with a maximum of three doses. Aseptic dispensing was performed in the Trasis HS700 isolator, a laminar flow cabinet designed for sterile handling.

### Study procedures

Patients received ¹⁷⁷Lu-PSMA-I&T at 6- to 8-week intervals for a total of three to six cycles. After the third cycle, an ¹⁸F-PSMA-PET scan was performed. Treatment response was assessed in a MDT meeting by integrating clinical and laboratory findings with imaging results. In the absence of biochemical or imaging evidence of disease progression, patients were allowed to continue treatment for up to six cycles. In the case of disease progression, the patient exited the study and transitioned to either a new line of treatment or palliative care.

Patients received 500 mL iv fluids (Plasmalyte®) and 8 mg of ondansetron orally 30 min before administration of ^177^LuPSMA-I&T, which was administered intravenously over 30 to 40 s. The planned flat dose was 7.4 GBq. No specific measures were taken to minimise xerostomia.

Safety reviews were conducted every 2 and 4-weeks after RLT with blood tests (complete blood count, kidney and liver function tests, lactate dehydrogenase and PSA) and either remote contact or outpatient clinic visit after each ^177^LuPSMA-I&T cycle. EORTC Quality of Life Questionnaire for cancer patients (EORTC-QLQ-C30, version 3.0) [25], prostate specific questionnaire (EORTC-QLQ-PR25) [26] and the Brief Pain Inventory (BPI short form) [27] was collected as a Health-related quality of life (HRQoL) on treatment days and 8 weeks after the last dose. AEs were evaluated at every contact according to Common Terminology Criteria for Adverse Events (CTCAE) v5.0.

To monitor and evaluate absorbed doses to kidneys and salivary glands, all patients underwent quantitative SPECT/CT scans after each therapy cycle. Dosimetry scans were performed at 4 and 24 h post-therapy, and if healthy-tissue uptake was high at 24 h timepoint additional 48-h time point was added. Absorbed doses were calculated according to the Medical Internal Radiation Dose (MIRD) formalism and reported after each cycle. In addition cumulative absorbed dose up to that point and estimated absorbed dose for six cycles was monitored. Maximum allowable doses for kidney and salivary glands were determined according to the European Association of Nuclear Medicine (EANM) procedure guideline [21].

### Statistical analysis

Study data were collected with the REDCap electronic data capture tool [28]. Statistical analysis was performed with IBM SPSS Statistics version 29.0.0.0. Survival analyses were conducted with the Kaplan Meyer method and log rank was used for determining statistical difference in univariant analysis. Univariate analysis was chosen because of the small sample size. All the statistical analysis were conducted with guidance of a KUH statistician.

## Results

### Patient characteristics

Two of the 40 patients withdrew their consent during the trial; therefore, the final analysis included 38 patients. The first patient was recruited on 23 FEB 2021 and the last patients last therapy was given on 18 OCT 2023. Patients received a median of 3 cycles (1–6 cycles) of ^177^Lu-PSMA-I&T. The mean administered activity was 7.38 GBq (standard deviation [SD] 0.19). Dosimetry scans were performed for all patients and the median cumulative dose for kidneys and salivary glands were 28.8 Gy and 5.1 Gy, respectively. Patient characteristics are presented in Table 1.

**Table 1:** Patient characteristics.

**Median age (range) – years**	71 (55–84)
	**Number of cases (*N*), %**
**ECOG performance status**	***N* (%)**
0	19 (50%)
1	14 (36.8%)
2	5 (13.2%)
Charlson comorbidity score (range)	9 (7–13)
**ISUP grade at diagnosis**	***N* (%)**
7	10 (26%)
8–10	27 (71%)
Unknown	1 (3%)
Synchronous metastasis Metachronous metastasis	18 (47%) 20 (53%)
**Metastatic sites at screening**	***N* (%)**
Bone metastases	0: 2 (5%)
	1–10: 9 (24%)
	11–20: 5 (13%)
	> 20: 22 (58%)
LN local	32 (84%)
LN other than local	36 (95%)
Visceral metastases	6 (15.8%)
**Previous treatments**	***N* (%)**
Docetaxel mCRPC	14 (37.8%)
Abiraterone	11 (28.9%)
Enzalutamide	29 (76.3%)
Cabazitaxel	13 (34.2%)
Radium-223	2 (5.3%)
**Previous systemic therapy lines**	***N* (%)**
1	5 (13.2%)
2	21 (55.2%)
3	11 (28.9%)
4	1 (3%)
Bone protecting agents (denosumab)	24 (63%)
**Local treatments**	***N* (%)**
Prostatectomy	4 (11%)
Curative radiotherapy	21 (55%)
Brachytherapy (LDR)	1 (3%)
Palliative RT to prostate	8 (21%)
Palliative RT to metastasis	25 (66%)
**Screening blood results**	***N* (%)**
Hemoglobin (g/L)	124 (93–154)
Platelets (10⁹/L)	256 (58–559)
WBC (10⁹/L)	6.5 (2.4–11.2)
GFR (ml/min)	85 (51–110)
ALP (U/L)	141 (43–846)
PSA (µg/L)	111 (7.1–849)
**¹⁷⁷Lu-PSMA-I&T treatment summary**	
Number of cycles	3 (range 1–6)
Mean dose	7.38 GBq (SD 0.19)
Kidney absorbed dose	28.8 Gy (1.7–202.90^a^ Gy)
Salivary gland absorbed dose	5.1 Gy (0.2–8.1 Gy)

a In one patient, a large adrenal gland metastasis interfered with the first-cycle dosimetry, resulting in an inaccurate absorbed kidney dose of 180.2 Gy. After applying three time-point dosimetry in later cycles (five cycles), the cumulative dose decreased to 20 Gy.

ECOG: Eastern Cooperative Oncology Group; mHSPC: metastatic hormone-sensitive prostate cancer; mCRPC: metastatic castration-resistant prostate cancer; NSAID: non-steroidal anti-inflammatory drug; RT: radiotherapy; LDR; low-dose-rate; GFR: glomerular filtration rate; ALP: alkaline phosphatase; LDH: lactate dehydrogenase; PSA: prostate-specific antigen; WBC: white blood cell; BHA: bone health agents; LN: lymph node; SD: standard deviation; Gy: grey (unit of absorbed radiation dose); GBq: gigabecquerel.

### Efficacy

**Figure 1:**
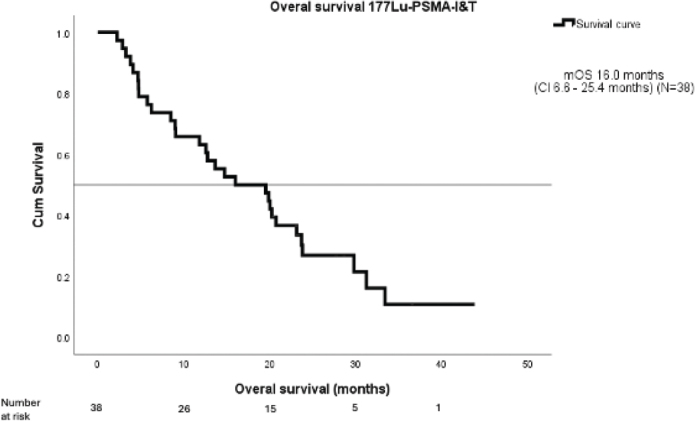
Overall survival ^177^LuPSMA-I&T – mOS.

**Figure 2:**
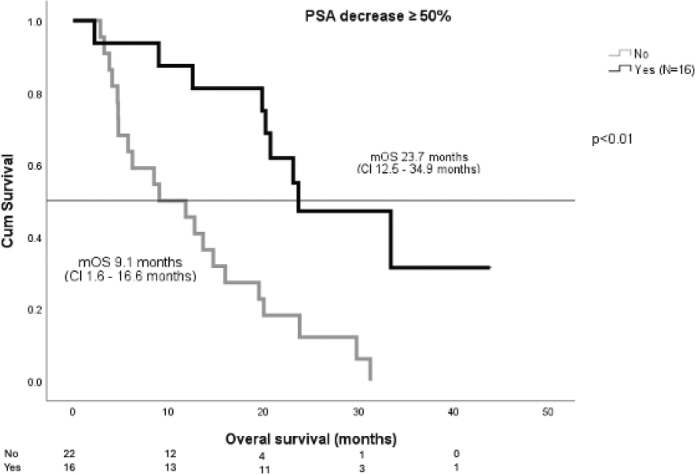
PSA decrease ≥ 50% – mOS.

**Figure 3:**
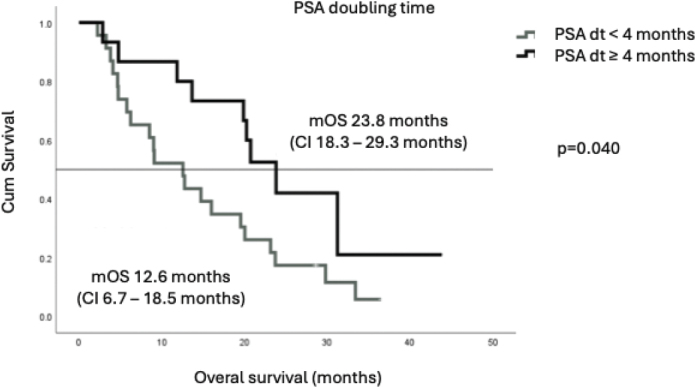
PSA doubling time – mOS.

mOS was 16.0 months (CI 6.6–25.4 months) (Figure 1). Patients with ECOG 0–1 had better mOS than those with ECOG 2 (20.0 vs 4.7 months, respectively; *p* < 0.01). A PSA decrease of 50% or more was seen in 16 patients (40%) and among those patients the mOS was significantly longer than in others (23.7 months vs. 9.1 moths, *p* < 0.01) (Figure 2). PSA doubling time (PSA dt) at screening was 4.2 months and was found to be predictive for treatment response. mOS for patients with PSA dt over 4 months was 23.8 months compared to 12.6 months among patients with PSA dt under four months (*p* = 0.040) (Figure 3). Visceral metastases were identified in six patients – one with both pulmonary and adrenal gland metastases, and five with adrenal gland metastases only. mOS in this subgroup was 18.4 months.

**Figure 4:**
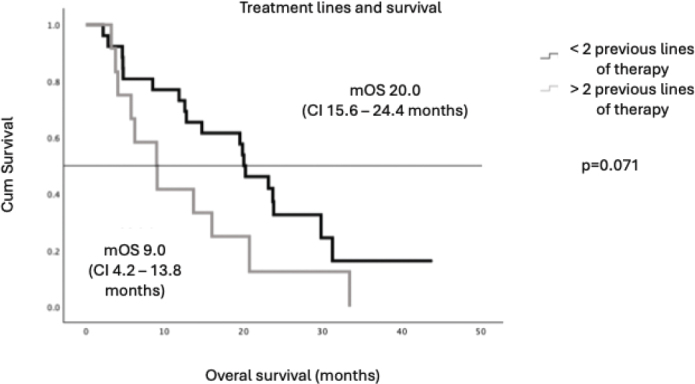
Treatment lines and survival.

**Figure 5:**
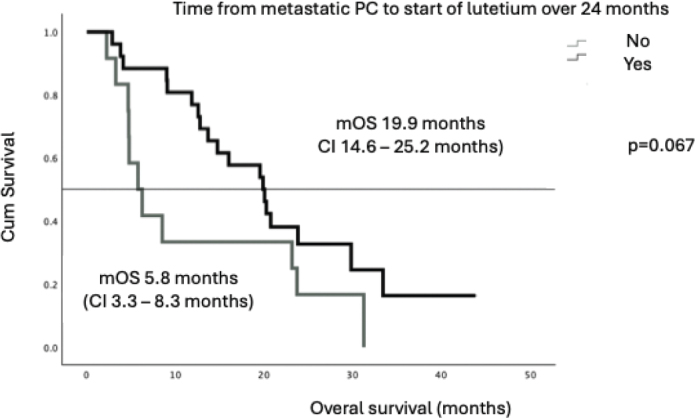
Time from metastatic PC to start of lutetium – mOS.

There was a trend towards longer survival among the patients whose time from primary diagnosis to metastatic disease exceeded 24 months (mOS 19.9 months vs. 5.8 months) (*p* = 0.067), although this was not statistically significant (Figure 4). Patients who received study treatment at second- or third-line therapy tended to have longer mOS compared to those treated in fourth or fifth line (20.2 months vs. 11.5 months respectively) (Figure 5). With metachronous metastases mOS was 20.3 months and with synchronous metastases only 12.8 months, but the difference was not statistically significant (*p* = 0.421).

### Adverse events

AEs were reported in 95% of the patients. Most of the AEs were grade 1 or 2. The incidence of grade 3 or higher AEs was very low. Only 12.3% of treated patients experienced grade 3 or higher AE which were mostly laboratory findings (anaemia, thrombocythemia, lymphopenia), infections (one pneumonia) or physical fitness deuteriation (two patients). One patient died from pneumonia following a single cycle of ¹⁷⁷Lu-PSMA; the event was not considered treatment-related. Xerostomia, a prespecified AE of interest occurred in 7 (18%) of the patients. No grade 3 or higher xerostomia AE was observed (Table 2).

**Table 2:** Adverse events.

Adverse event	Grades 1–2 (%)	≥ Grade 3 number (%)
Anaemia	19 (50%)	1 (2.6%)
Lymphocytopenia	9 (23.7%)	3 (7.9%)
Thrombocytopenia	6 (15.8%)	3 (7.9%)
Physical fitness deterioration	1 (2.6%)	2 (5.3%)
Pneumonia	0 (0%)	1 (2.6%)*
Leukopenia	8 (21.1%)	-
Hypocalcaemia	8 (21.1%)	-
Dry mouth	7 (18.4%)	-
Nausea	6 (15.8%)	-
Fatigue	4 (10.5%)	-
Pain	4 (10.5%)	-
Neutropenia	1 (2.6%)	-
Infection NAS	1 (2.6%)	-
Creatinine increased	1 (2.6%)	-
Impaired renal function	1 (2.6%)	-

NAS: not otherwise specified.

* there was one grade 5 pneumonia after one cycle of lutetium which was deemed not related to treatment based on otherwise normal blood results.

## Discussion and conclusion

The KuPSMALu trial is the first RWE study in the Nordic countries assessing the efficacy and tolerability of in-house produced ^177^Lu-PSMA-I&T in patients with mCRPC who had previously been treated with chemotherapy and at least one ARPi and received treatment within the public healthcare system. The most significant benefit from the ^177^Lu-PSMA therapy was achieved among the patients with ECOG 0-1, the PSA dt over 4.2 months and a PSA decrease of 50% or more.

In the entire study population, the mOS was 16.0 months, closely aligning with the results of the landmark VISION and TheraP trials, where mOS was 15.3 and 19.1 months, respectively. The efficacy of ^177^Lu-PSMA has been evaluated in other real-world studies with mixed results. Ling S. et al. reported RWE for patients treated with two lines of cabazitaxel and at least one line of ARPi with an mOS of 8.1 months [29]. In the large European retrospective RWE study and PACAP study, mOS was reported to be 13.7 months [30]. The inferiority of these results, compared to ours, may be due to patient selection, exclusion criteria for PSMA-negative disease and the different ^177^Lu-PSMA ligands used. These findings demonstrate that, even with in-house production of ¹⁷⁷Lu-PSMA-I&T, the KuPSMALu trial achieved outcomes consistent with internationally validated standards for treating mCRPC.

Notably, a mOS exceeding 20 months was observed among patients with an ECOG performance status of 0–1, a PSA dt longer than 4.2 months and those experiencing a ≥ 50% decline in PSA levels. In addition, a favourable trend in mOS – also exceeding 20 months – was seen in subgroups including patients with a time interval of more than 2 years from primary diagnosis to metastatic disease, those with metachronous metastases and those who received ¹⁷⁷Lu-PSMA therapy as a second- or third-line treatment. Although improved outcomes are typically observed in patients with good performance status, the difference between the subgroups exceeded expectations. The outcomes among patients with ECOG 2 were clearly inferior (mOS 4.7 months), indicating that poor performance status may predict limited benefit from ¹⁷⁷Lu-PSMA-I&T. However, since only five patients in our cohort had ECOG 2, this observation should be interpreted with caution. In clinical practice, RLT should preferably be offered to patients with ECOG 0–1 whenever feasible. Similarly, patients with minimal PSA decline had an mOS of 9.1 months, and those with a short PSA dt experienced an mOS of just 12.6 months.

Tolerability in KuPSMALu was favourable, with grade ≥ 3 AEs in only 12.3% of patients – comparable with the VISION (15%) and PACAP (17%) studies [23, 30]. This low incidence of severe toxicity emphasizes the safety of in-house produced ¹⁷⁷Lu-PSMA-I&T and supports its broader integration into public healthcare settings. Xerostomia, a common AE associated with salivary gland uptake, occurred in 18% of patients, none of grade ≥ 3. In comparison, xerostomia was observed in 38.8% and 15% in the VISION trial and PACAP trial respectively [23, 30]. Dosimetry results for the salivary glands and kidneys were in line with EANM guidelines [21].

A consistent pattern was detected in this study: while some patients derived an exceptionally robust and durable benefit from ¹⁷⁷Lu-PSMA therapy, others experienced rapid disease progression shortly after treatment initiation. This variability in treatment response may be driven by tumour evolution towards a more aggressive phenotype – such as neuroendocrine differentiation – or by underlying molecular alterations that promoting resistance. For example, genomic alterations in the androgen receptor (AR) and PI3K pathways detected from circulating tumour DNA (cfDNA) have been associated with inferior responses to ¹⁷⁷Lu-PSMA-I&T [31]. This supports the well-established association between PSA doubling time and tumour aggressiveness – a factor that can be used to enhance future patient selection for PSMA-targeted RLT. Looking ahead, it will be essential to identify additional predictive biomarkers that can further refine treatment stratification and optimise outcomes. In the interim, a deep PSA decline (≥ 50% reduction) remains a simple, cost-effective and accessible early indicator of treatment response, and may inform decisions regarding treatment continuation.

Different approaches to assessing PSMA-positive versus PSMA-negative disease have been used in earlier trials. In the VISION trial, patients were screened with ^68^Ga-PSMA-PET and body CT, while the TheraP trial used ^68^Ga-PSMA-PET and FDG-PET to exclude patients with PSMA negative disease [23, 24]. In the KuPSMALu trial, ¹⁸F-PSMA PET and contrast-enhanced CT were used to select only PSMA-positive patients. The omission of FDG-PET reduced the cost of pre-treatment evaluation without compromising OS. Financial toxicity remains one of the main barriers to the wider adoption of ¹⁷⁷Lu-PSMA therapies. High demand, shortages of ¹⁷⁷Lu, and regulatory requirements for radiopharmaceuticals all contribute to the cost of commercial ¹⁷⁷Lu-PSMA-617. In contrast, in-house production of ¹⁷⁷Lu-PSMA-I&T reduced the financial burden while maintaining efficacy. While most mCRPC patients present with bone-predominant disease, advanced imaging such as ¹⁸F-PSMA PET shows that nearly all have at least one extra-skeletal lesion. Unlike radium-223, ¹⁷⁷Lu-PSMA can target both bone and soft tissue metastases, offering a more comprehensive therapeutic approach.

When the study protocol for the KuPSMALu trial was created, cabazitaxel was still under patent in Finland, making in-house ^177^LuPSMA-I&T a financially reasonable choice over cabazitaxel. Nowadays the financial issues have shifted in favour of cabazitaxel, but the better tolerability of ^177^Lu-PSMA-I&T still applies. Prostate cancer patients tend to be older and therefore the side-effects of the chosen therapy are an important factor. With appropriate patient selection, ^177^Lu-PSMA-I&T is well-tolerated, and its impact on performance status is minimal, corresponding with favourable treatment outcomes.

RLTs are a rising field in oncology. ^177^Lu-PSMA was the first PSMA targeted RLT to receive FDA and EMA approval. At present, an increasing number of isotopes and targets are under investigation. The most advanced among these is ^225^Ac-PSMA-directed RLTs. Compared with ^177^Lu, a beta-emitter, ^225^Ac is an alpha emitter, whose decay scheme is more energetic than that of beta emitters [32]. This enables more DNA double-strand breaks with fewer hits. The effect of ^225^Ac-PSMA RLT has been shown to be effective in the retrospective WARMTH Act trial [32]. When comparing results of the KuPSMALu and PACAP trials with those of the WARMTH Act trial, it appears that with ^225^Ac-PSMA RLT is associated with more xerostomia (including grade 3) than ^177^Lu-PSMA [32].

In the KuPSMALu trial, patients were required to have at least one metastatic lesion outside the skeletal system. While radium-223 remains an effective therapeutic option for patients with bone-only metastatic disease, its lack of activity against visceral and nodal metastases limits its use in more disseminated cases. Similar to the findings reported, where ¹⁷⁷Lu-PSMA radioligand therapy demonstrated efficacy in isolated bilateral adrenal metastases, our results also suggest that ¹⁷⁷Lu-PSMA-I&T is effective in patients with adrenal gland involvement [33].

The KuPSMALu trial demonstrated that in-house production of ¹⁷⁷Lu-PSMA-I&T, combined with patient selection through ¹⁸F-PSMA-PET and contrast-enhanced CT, provides an effective and cost-efficient treatment strategy for patients with PSMA-positive mCRPC, with minimal serious adverse effects. A statistically significant survival benefit was observed particularly among patients with a PSA doubling time exceeding 4 months, highlighting the importance of biological disease kinetics in predicting treatment response. Prostate cancer remains one of the most prevalent malignancies in the Western world. Although RLT offers promising outcomes, it is associated with high costs and requires specialised infrastructure and expertise. Optimal patient selection is crucial to maximise therapeutic benefit and resource efficiency. Further studies are needed to define the optimal sequencing of ¹⁷⁷Lu-PSMA-I&T within the broader treatment landscape of mCRPC and to identify robust predictive biomarkers that can guide individualised treatment decisions.

## Supplementary Material



## Data Availability

The data are not publicly available due to privacy and ethical restrictions. The data that support the findings of this study may be available from the corresponding author on reasonable request and with required permissions.
